# Co-inhibition of mTORC1, HDAC and ESR1α retards the growth of triple-negative breast cancer and suppresses cancer stem cells

**DOI:** 10.1038/s41419-018-0811-7

**Published:** 2018-07-26

**Authors:** Andrew Sulaiman, Sarah McGarry, Ka Mien Lam, Sara El-Sahli, Jason Chambers, Shelby Kaczmarek, Li Li, Christina Addison, Jim Dimitroulakos, Angel Arnaout, Carolyn Nessim, Zemin Yao, Guang Ji, Haiyan Song, Sheng Liu, Ying Xie, Suresh Gadde, Xuguang Li, Lisheng Wang

**Affiliations:** 1 Department of Biochemistry, Microbiology and Immunology, Faculty of Medicine, University of Ottawa, 451 Smyth Road, Ottawa, ON K1H 8M5 Canada; 2 China-Canada Centre of Research for Digestive Diseases, University of Ottawa, 451 Smyth Road, Ottawa, ON K1H 8M5 Canada; 3 Institute of Digestive Diseases, Longhua Hospital, Shanghai University of Traditional Chinese Medicine, 725 South Wanping Road, 200032 Shanghai, China; 4 Centre for Infection, Immunity and Inflammation, Faculty of Medicine, University of Ottawa, Ottawa, Canada; 5 Ottawa Institute of Systems Biology, University of Ottawa, 451 Smyth Road, Ottawa, ON K1H 8M5 Canada; 6 Centre for Cancer Therapeutics, Ottawa Hospital Research Institute, Ottawa, ON K1H 8L6 Canada; 7 Institute of Chinese Traditional Surgery, Longhua Hospital Affiliated to Shanghai University of Traditional Chinese Medicine, 725 South Wanping Road, 200032 Shanghai, China; 8 Centre for Biologics Evaluation, Biologics and Genetic Therapies Directorate, Health Canada Sir Frederick G. Banting Research Centre, A/L 2201E, 251 Sir Frederick Banting Driveway, Ottawa, ON K1A 0K9 Canada; 9 Regenerative Medicine Program, Ottawa Hospital Research Institute, Ottawa, ON K1H 8L6 Canada

## Abstract

Triple-negative breast cancer (TNBC) is the most refractory subtype of breast cancer. It causes the majority of breast cancer-related deaths, which has been largely associated with the plasticity of tumor cells and persistence of cancer stem cells (CSCs). Conventional chemotherapeutics enrich CSCs and lead to drug resistance and disease relapse. Development of a strategy capable of inhibiting both bulk and CSC populations is an unmet medical need. Inhibitors against estrogen receptor 1, HDACs, or mTOR have been studied in the treatment of TNBC; however, the results are inconsistent. In this work, we found that patient TNBC samples expressed high levels of mTORC1 and HDAC genes in comparison to luminal breast cancer samples. Furthermore, co-inhibition of mTORC1 and HDAC with rapamycin and valproic acid, but neither alone, reproducibly promoted ESR1 expression in TNBC cells. In combination with tamoxifen (inhibiting ESR1), both S6RP phosphorylation and rapamycin-induced 4E-BP1 upregulation in TNBC bulk cells was inhibited. We further showed that fractionated CSCs expressed higher levels of mTORC1 and HDAC than non-CSCs. As a result, co-inhibition of mTORC1, HDAC, and ESR1 was capable of reducing both bulk and CSC subpopulations as well as the conversion of fractionated non-CSC to CSCs in TNBC cells. These observations were partially recapitulated with the cultured tumor fragments from TNBC patients. Furthermore, co-administration of rapamycin, valproic acid, and tamoxifen retarded tumor growth and reduced CD44^high/+^/CD24^low/^^−^ CSCs in a human TNBC xenograft model and hampered tumorigenesis after secondary transplantation. Since the drugs tested are commonly used in clinic, this study provides a new therapeutic strategy and a strong rationale for clinical evaluation of these combinations for the treatment of patients with TNBC.

## Introduction

Breast cancer is one of the leading causes of cancer-related deaths in women throughout the world^1^. The triple-negative breast cancer (TNBC) subtype is characterized as being negative for the estrogen receptor 1 (ESR1), progesterone receptor (PGR), and human epidermal growth factor receptor type 2 (HER2). TNBC patients have high rates of recurrence between the first and third year of treatment, with the majority of deaths occurring within the first 5 years^2,3^. It is one of the most difficult subtypes of breast cancer to treat and disproportionately causes the majority of breast cancer-related deaths^4^.

Because of the lack of specific targets, chemotherapy regimens are a mainstay for TNBC treatment. Chemotherapeutics, however, have been shown to enrich cancer stem cells (CSCs) in TNBC^5–7^. These CSCs (e.g., CD44^high/+^/CD24^low/−^ subpopulation) have been shown to regenerate the heterogeneous tumor in vivo, promoting chemoresistance, and disease relapse^6,8^. Owing to tumor plasticity and the conversion between CSC and non-CSC subpopulations^9–12^, development of a strategy capable of inhibiting both non-CSC and CSC subpopulations is crucial for TNBC therapy^13^.

Given the excellent efficacy-to-toxicity ratio of anti-ESR1 treatment, functional reactivation of ESR1 by inhibition of phosphoinositide 3 kinase (P13K)/Akt/mammalian target of rapamycin complex 1 (mTORC1) signaling or histone deacetylase (HDAC) to sensitize TNBC to endocrine therapy has been explored but with inconsistent results and undefined mechanisms^14^.

The P13K/Akt/mTORC1 pathway is commonly activated in breast cancer. For example, phosphatase and tensin homolog, the negative regulator of P13K, is mutated at a frequency of 44% in luminal and 67% in TNBC^15^, leading to both endocrine and chemotherapeutic resistance^16–18^. It has been shown that P13K/Akt/mTORC1 activation induces estrogen-independent ESR1 signaling to promote endocrine resistance^19^. P13K/Akt/mTORC1 activation also affects the epigenetic regulation of the chromatin. It modifies histone methylation, acetylation, and ubiquitination, resulting in the aberrant silencing/repression of various genes^20–22^. However, using mTORC1 inhibitors alone failed in the treatment of several types of tumor^23–25^. This has been attributed to incomplete inhibition of mTORC1. mTORC1 signaling consists of S6RP phosphorylation and eukaryotic translation initiation factor 4E-binding protein 1 (4E-BP1) phosphorylation that stimulates cap-dependant translation. Rapamycin demonstrates a high affinity of inhibition toward S6K1 phosphorylation, but it induces 4EBP1-phosphorylation within 6 h of treatment, allowing for cap-dependant translation and mTORC1 signaling^26^. As such, suppressing both S6RP and 4E-BP1 phosphorylation is required for a viable mTORC1 inhibition.

HDACs have been shown to epigenetically suppress ESR1^27,28^. As such, HDAC inhibitors have been tested to promote ESR1 re-expression in TNBC. Preclinical studies have shown that various HDAC inhibitors (e.g., PCI-24781, trichostatin A, valproic acid, and vorinostat) in combination with tamoxifen (a selective estrogen receptor (ER) modulator) lead to endocrine sensitivity and increased cell death of breast cancer. However, these results are controversial with undefined mechanisms^29–34^.

In this study, we observed that tumor samples from TNBC patients expressed higher levels of mTORC1 and HDAC genes than those from non-TNBC luminal breast cancer. The fractionated TNBC CSC subpopulation expressed higher levels of mTORC1 and HDAC mRNA than non-CSCs. Accordingly, the combination of low dose of rapamycin (repressing mTORC1/S6RP) and valproic acid (a pan HDAC inhibitor) restored ESR1 expression; the combination of rapamycin, valproic acid, and tamoxifen suppressed both S6RP and 4E-BP1 phosphorylation and effectively repressed both bulk and CSC subpopulations in TNBC. Furthermore, in a human xenograft model, three inhibitors in combination effectively attenuated TNBC tumor burden, diminished the CD44^high/+^/CD24^low/^^−^ CSC subpopulation, and reduced tumorigenesis after secondary transplantation. Combination pharmacologic therapies have been proposed as one of the most promising strategies in breast cancer studies^35^. These findings suggest that co-inhibition of mTORC1, HDAC, and ESR1 can be considered as a tangible approach to target both TNBC bulk and CSC populations in a clinical setting.

## Materials and methods

### Cell culture and reagents

SUM149-PT breast cancer cells were obtained from Asterand (Detroit, MI, USA) and cultured in Hams F-12 media (Mediatech, Manassas, VA, USA) containing 5% fetal bovine serum (FBS), 5 μg/ml insulin, 1 μg/ml hydrocortisone, 10 mM HEPES, and 1% penicillin/streptomycin. MDA-MB-231 breast cancer cells were purchased from the American Type Culture Collection (ATCC, Manassas, VA, USA) and maintained in Dulbecco’s modified Eagle’s medium-F12 media supplemented with 10% FBS (HyClone, Logan, UT, USA) and 1% penicillin/streptomycin. Cells were cultured at 37 °C in a 5% CO_2_ incubator. Tamoxifen was purchased from CalBiotech (El Cajon, CA, USA), rapamycin from Caymen Chemicals (Ann Arbor, Michigan, USA), and valproic acid from Sigma (Oakville, ON, Canada). Insulin, hydrocortisone, HEPES, and bovine serum albumin were purchased from Sigma-Aldrich (St. Louis, MO, USA).

### Breast cancer tissue and patient-derived xenograft fragments

Tumor tissues from three TNBC patients undergoing routine surgical procedures were obtained. The protocol was approved by The Ottawa Hospital Research Ethics Board (Protocol# 20120559-01H). Approximately 2 mm cores were obtained using a sterile biopsy punch that was further sliced with a scalpel to obtain approximately 2 × 1 mm^2^ tumor slices^9,11,36^. The slices were randomized and three slices were placed into each well of 24-well plate and cultured in DMEM-F12 medium supplemented with 10% FBS, 1% penicillin/streptomycin, 1 µg/ml insulin, 0.5 ng/ml hydrocortisol, and 3 ng/ml epidermal growth factor. These primary tissue fragments were treated with the same concentrations of inhibitors as described in the figures, followed by a viability assay and flow cytometric analysis. The TNBC patient-derived xenograft sample HCI-001 was obtained from University of Utah and cultured in the same conditions as the clinical samples.

### Flow cytometric analysis

Dissociated cancer cells were filtered through a 4 µm strainer and suspended in phosphate-buffered saline (PBS) supplemented with 2% FBS and 2 mM EDTA (fluorescence-activated cell sorting (FACS) buffer) as previously described^11^. In all, 1 µL of mouse IgG (1 mg/mL) was added and incubated at 4 °C for 10 min. The cells were then re-suspended in 1× binding buffer and anti-CD44 (allophycocyanin) in combination with anti-CD24 (phycoerythrin (PE)) (BD, Mississauga, ON, Canada) antibodies were added according to the manufacturer’s instructions. The cells were washed twice with FACS buffer and 7-aminoactinomycin D (7-AAD, eBioscience, San Diego, CA) and Annexin-V/PE-Cy7 (eBioscience) was added and incubated for 15 min at room temperature to assess dead and apoptotic cells. Flow cytometry was performed on a Cyan-ADP 9 and the BD LSRFortessa. Data was analyzed with the FlowJo software (Ashland, OR, USA).

### Fractionation of CSC and non-CSC subpopulations from breast cancer cells

CSCs and the bulk populations were separated based on CD44^high/+^/CD24^low/−^ expression in MDA-MB-231 cells^11^. After antibody staining, four subpopulations were analyzed and sorted by MoFlo Astrios Sorter (Beckman Coulter). Isolation gates, including histogram markers and dot plot quadrants, were chosen based on negative controls. Purity (>90%) was determined after sorting.

### Western blot analysis

Cells were harvested, washed with PBS, and lysed with lysis buffer supplemented with protease inhibitors (Roche, Sainte-Agathe-Nord, QC, Canada). Protein concentrations were determined using a Bio-Rad DC Protein Assay Kit (Bio-Rad, Hercules, CA, USA), and samples were then normalized. The samples were loaded into an 8–10% polyacrylamide gel and separated by sodium dodecyl sulfate-polyacrylamide gel electrophoresis followed by transference to a polyvinylidene difluoride membrane. Proteins were identified by incubation with primary antibodies followed by horseradish peroxidase-conjugated secondary antibodies and an enhanced chemiluminescence solution (Pierce, Thermo Scientific, Waltham, MA, USA). Antibodies used in this study include: anti-phosphorylated S6 Ribosomal Protein (1:1000, Cat: 2211s, Cell Signaling, Cambridge, MA, USA), anti-S6 Ribosomal Protein (8E2) monoclonal antibody (1:1000, Cat: 2217s, Cell Signaling), anti-4E-EBP1 (1:1000, Cat: 9452s, Cell Signaling), anti-phosphorylated 4E-BP1 (1:1000, Cat: 2855s, Cell Signaling), anti-acetylated Histone 3 (1:1000, Cat: 4243s, Cell Signaling), anti-Histone 3 1:1000, Cat: 9715s, Cell Signaling), anti-ESR1α (1:1000, Cat: MCA1799T, Bio Rad, CA, USA), and anti-α-tubulin monoclonal antibody (1:500, Cat: T9026, Sigma-Aldrich, St. Louis, MO, USA).

### Quantitative real-time PCR (RT-qPCR)

Total RNAs were extracted using the RNeasy Kit (QIAGEN) and RT-qPCR analysis was performed using Bio-Rad MyiQ (Bio-Rad, Hercules, CA, USA) as previously described^9,11^. The conditions for RT-qPCR reactions were: one cycle at 95 °C for 20 s followed by 45 cycles at 95 °C for 3 s and annealing at 60 °C for 30 s. Results were normalized to the housekeeping gene 18s ribosomal RNA (18s) or glyceraldehyde 3-phosphate dehydrogenase. Relative expression level of genes from different groups were calculated with the 2ΔΔCT method and compared with the expression level of appropriate control cells. Specific primer sequences for individual genes are listed in Supplemental Table S1.

### Small interfering RNA (siRNA) knockdown

siRNAs for S6RP (#AM16708) and the Silencer Select Negative Control #1 siRNA (Scramble, #4390843) were purchased from Thermo Scientific (Waltham, MA,, USA) as SMARTpools. For siRNA transfections, cells were transfected with oligos using Lipofectamine RNAiMAX reagent (Invitrogen, Carlsbad, CA, USA) according to the manufacturer’s instructions. After transfection, efficiency was determined through western blot or RT-qPCR.

### Cell viability assays

Cells were seeded into 12-well plates (1.5 × 10^4^ cells/well). After 120 h of treatment, Alamar blue viability analysis was performed by incubation with 10% Alamar blue reagent (Thermo Fisher Scientific) for 4 h. Florescence was measured at 560 nm excitation and 590 nm emission. Cell viability was also determined through 3-(4,5-dimethylthiazol-2-yl)-2,5-diphenyl tetrazolium bromide (MTT, 5 mg/ml) staining after incubation for 4 h. Absorbance was measured at 570 nm.

### Xenograft tumor growth

Athymic nude mice were obtained from Charles River Laboratories. The SUM149-PT or MDA-MB-231 breast cancer cells were mixed in 1:1 ratio with Matrigel and injected under aseptic conditions into the mammary fat pads (*n* = 4 for each group, 2.5 × 10^6^ cells per fat pad). When the tumor reached a mean diameter of ~3 mm, mice were randomly divided into two groups and intraperitoneally injected daily with the vehicle (dimethyl sulfoxide (DMSO)) or valproic acid (300 mg/kg/day)+rapamycin (1.5 mg/kg/day)+tamoxifen (0.4 mg/kg/day) for 20 days. At the end of treatment, mice were humanely euthanized and tumors were harvested for further analyses and secondary transplantation.

### Secondary transplantation to assess cancer-initiating capacity

Tumors were minced using a scalpel and incubated in antibiotic-free DMEM media containing collagenase/hyaluronidase (STEMCELL Technologies, #07912) at 37 °C. Dissociated single cells were collected every 15 min while tumor fragments were digested further to obtain single cells^9^. Afterwards, the cells were passed through a 40 µM nylon mesh. The dissociated tumor cells were inoculated into one of the mammary fat pads at a concentration of 10^5^, 10^4^, 10^3^, or 10^2^ cells from the original tumors. Tumor growth and size were measured after 6 weeks of growth.

### Clinical database analysis and statistical analysis

Breast cancer datasets from The Cancer Genome Atlas (TCGA, http://cancergenome.nih.gov/) were analyzed with cBioportal (http://www.cbioportal.org/index.do)^37,38^. High expression of HDAC gene was defined as mRNA expression levels >2.5 standard deviations above the mean. High expression of mTORC1 gene was defined as mRNA levels >2 standard deviations above the mean. Expression data and Kaplan–Meier survival curves were generated using datasets compiled by August 2017 from the following Database IDs (529 patients): mTORC1 and HDAC gene enrichment: http://bit.ly/2wgwyhy, mTORC1 gene enrichment: http://bit.ly/2wh8Mlz, and HDAC gene enrichment: http://bit.ly/2whb97U.

Gene Expression Omnibus2R database was used to analyze a dataset (Dataset: GSE65216) to compare TNBC cell lines to 55 TNBC patient samples (https://www.ncbi.nlm.nih.gov/geo/geo2r/?acc=GSE65216&platform=GPL570). For all clinical database data, the log-rank test was performed to determine whether observed differences between groups were statistically significant. Statistical significance was determined via adjusted *P* values using Benjamini and Hochberg false discovery rate method by default. Results were considered significant when *p < 0.05, **p < 0.01, or ***p < 0.001.

## Results

### Tumor samples from TNBC patients express higher level of mTORC1 and HDAC than those of non-TNBC patients and are associated with decreased ESR1 expression and reduced survival rate

**Fig. 1:**
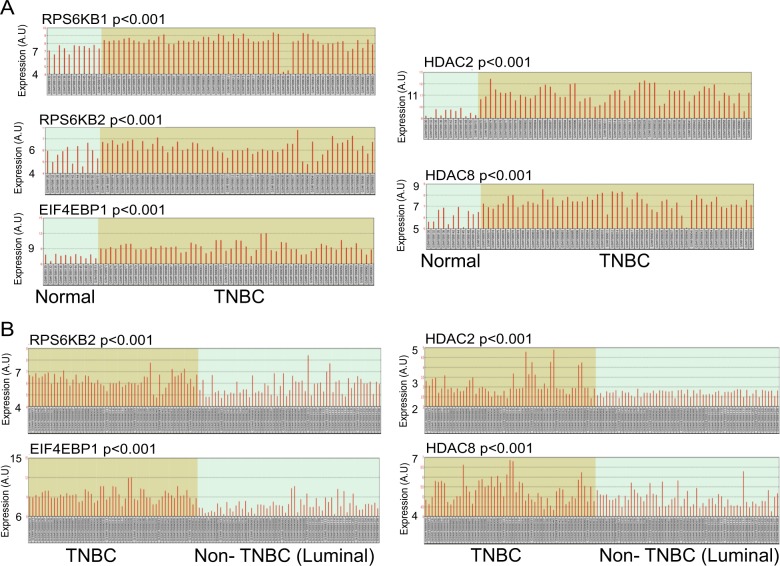
Gene expression levels of mTORC1 and HDAC are higher in TNBC tumor than in normal breast tissues and luminal A/B breast cancer. **a** The relative expression levels (A.U arbitrary unit) of mTORC1 and HDAC genes in 55 TNBC patient tumors and 11 normal breast tissue samples were compared using the NCBI Gene Expression Omnibus (GEO2R). The GSE65216 samples were analyzed with the Affymetrix Human Genome U133 Plus 2.0 Array (GPL570). **b** The relative expression levels (A.U: arbitrary unit) of mTORC1 and HDAC genes in 55 TNBC patient tumors and 59 luminal A/B breast cancer samples were compared using the NCBI Gene Expression Omnibus (GEO2R). The GSE65216 samples were analyzed with the Affymetrix Human Genome U133 Plus 2.0 Array (GPL570)

To determine the correlation between HDAC, mTORC1, and ESR1 in TNBC patients, we analyzed normal mammary tissue, TNBC, and luminal breast cancers (ESR1 positive), using samples from 55 TNBC, 59 luminal A/B breast cancer, and 11 normal breast tissues (gene omnibus2R platform, Dataset: GSE65216, Accessed November 1, 2017^39–43^). The data was obtained by transcriptome analysis (Affymetrix Human Genome U133 Plus 2.0 Array, GPL570). We found that TNBC samples expressed higher levels of mTORC1 and HDAC mRNAs than normal breast tissue (Fig. 1a) and luminal A/B samples (Fig. 1b). These results suggest that patients with TNBC might be sensitive to HDAC and mTORC1 inhibition.

**Fig. 2:**
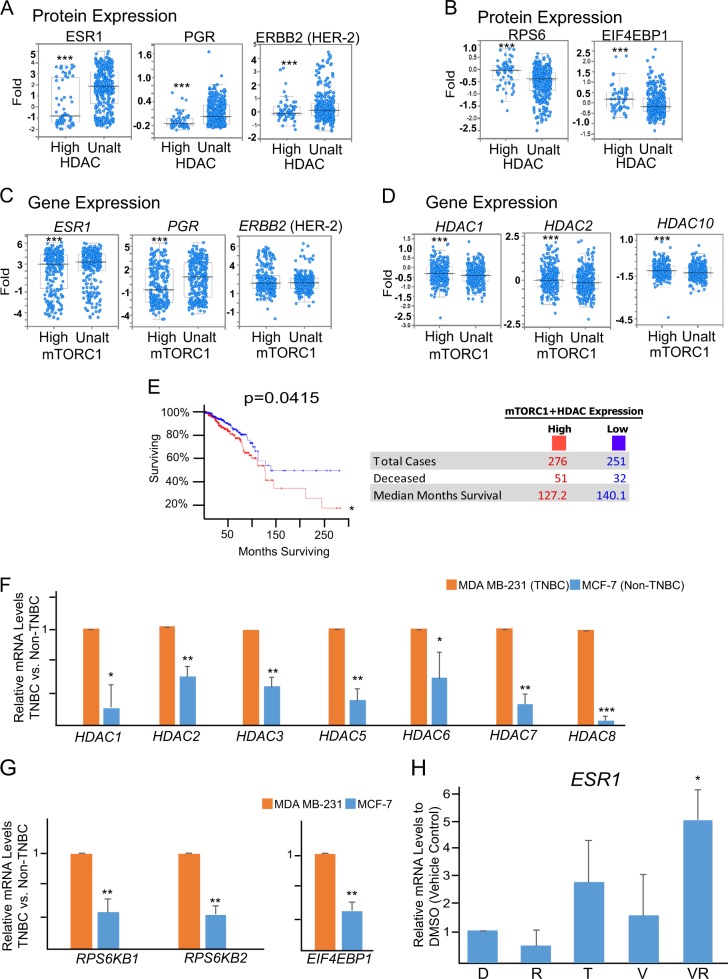
The expression levels of mTORC1 and HDAC are inversely associated with ESR1 and PGR in patients with invasive breast cancer and in TNBC cells. **a** Low expression (RRPA) of ESR1, PGR, and ERBB2 proteins in patients’ tumors inversely associated with high expression of HDAC target genes (*HDAC1*, *HDAC2*, *HDAC3*, *HDAC4*, and *HDAC10*) in comparison to their unaltered counterparts (Ctrl: control, *n* = 892 patients with invasive breast cancer, ***p < 0.001). **b** High expression (RRPA) of S6RP and EIF4EBP1 proteins in patients’ tumors positively associated with high expression of HDAC target genes (*HDAC1*, *HDAC2*, *HDAC3*, *HDAC4*, and *HDA*) in comparison to their unaltered counterparts (Ctrl: control, *n* = 892 patients with invasive breast cancer, ***p < 0.001). **c** Low expression (Microarray) of *ESR1*, *PGR*, and *ERBB2* in patients’ tumors inversely associated with high expression of mTORC1 target genes (*MTOR*, *MYC*, *CTSD*, *LDHA*, *MLST8*, *SCDP1*, *ACOX1*, *CPT1A*, *LSS*, *NRF1*, *TWIST1*, *SNAI1*, *TWIST2*, and *S6RPKB2*) in comparison to their unaltered counterparts (Ctrl: control, *n* = 892 patients with invasive breast cancer, ***p < 0.001). **d** High expression (Microarray) of HDAC target genes (*HDAC1*, *HDAC2*, and *HDAC3)* in patients’ tumors positively associated with high expression of mTORC1 target genes (see above) in comparison to their unaltered counterparts (Ctrl: control, *n* = 825 patients with invasive breast cancer, ***p < 0.001). **e** Kaplan–Meier survival curve for overall survival of the patients with high levels of mTORC1 and HDAC gene expression in cancer samples (red curve) in comparison to patients with unaltered expression (blue curve). *N* = 527, *p < 0.05, log-rank test. **f**–**g** RT-qPCR analysis and comparison of relative mRNA levels of HDAC (*HDAC1*, *HDAC2*, *HDAC3*, *HDAC5*, *HDAC6*, *HDAC7*, and *HDAC8*) and mTORC1 (*RPS6KB1*, *RPS6KB2*, and *EIF4EBP1*) genes between TNBC MDA-MB-231 and non-TNBC luminal breast cancer MCF-7 cell lines. **h** RT-qPCR analysis of the expression of ESR1 gene expression in TNBC MDA-MB-231 cells after treatment with DMSO (D) vehicle control, rapamycin (R, 5 nM), tamoxifen (T, 1 µM), valproic acid (V, 250 µM), or the combination of valproic acid and rapamycin (VR) for 120 h. mRNA levels are relative to the cells treated with DMSO vehicle control

We further analyzed a TCGA dataset containing 529 patients with invasive breast cancer (cBioportal)^37,38^ and found that the expression of HDAC protein was inversely correlated with the expression of ESR1 and PGR proteins. In contrast, the expression of HDAC protein was positively correlated with the expression of mTORC1-related S6RP and EIF4EBP1 proteins (Fig. 2a, b, Dataset ID: http://bit.ly/2whb97U). Also, elevated mTORC1 gene expression negatively associated with low levels of ESR1 and PGR gene expression, while elevated HDAC protein expression positively associated with elevated HDAC gene expression (Fig. 2c, d, Dataset ID: http://bit.ly/2wh8Mlz). Additionally, patients with low expression levels of both mTORC1 and HDAC mRNAs in their tumor samples exhibited an increased survival rate (Fig. 2e, Database ID: http://bit.ly/2wgwyhy, Supplemental Fig. 1a).

The trend observed in clinical datasets was also seen in breast cancer cell lines. Both HDAC and mTORC1 gene expressions were higher in MDA-MB-231 TNBC cells than in luminal ESR1+MCF-7 breast cancer cells (Fig. 2f, g). Accordingly, combination of 250 µM valproic acid (a pan-HDAC inhibitor) and 5 nM rapamycin (mTORC1 inhibitor), but neither alone, increased ESR1 gene expression in TNBC cells (Fig. 2h).

### Combination of mTORC1, HDAC, and ESR1 inhibitors restores ESR1 expression, suppresses rapamycin-induced 4E-BP1 upregulation, and inhibit TNBC cell viability

**Fig. 3:**
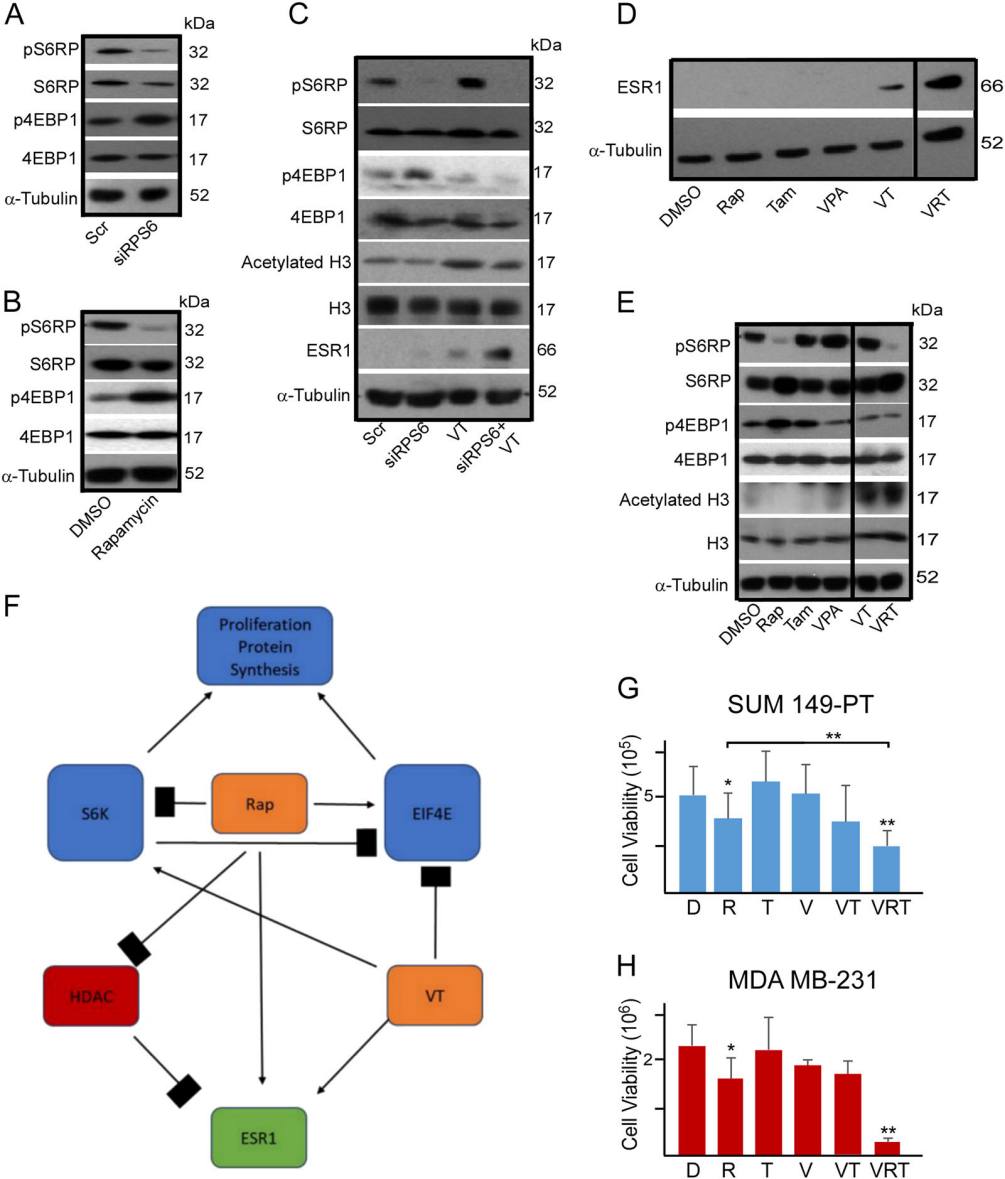
Co-inhibition of mTORC1, ESR1α, and HDACs restores ESR1 expression in TNBC cells and suppresses the expression of pRSP6, p4E-BP1, and HDAC and the growth of TNBC cells. **a** Representative western blot depicting S6RP and 4E-BP1 expression in MDA-MB-231 cells after knockdown of S6 ribosomal protein (siS6RP) in comparison to the scramble (Scr) control. **b** Representative western blot depicting S6RP and 4E-BP1 expression in MDA-MB-231 cells after rapamycin treatment (5 nM) in comparison to the vehicle (DMSO) control. **c** Representative western blot depicting S6RP, 4E-BP1, acetylated Histone H3, and ESR1 protein expression in MDA-MB-231 cells after knockdown of S6 ribosomal protein (siS6RP) in combination with valproic acid (VPA, 250 µM) and/or tamoxifen (Tam, 1 µM) for 48 h. VT: VPA+Tam. **d** Representative western blot depicting the ESR1 expression in MDA-MB-231 cells after combinational treatment with DMSO vehicle control; rapamycin (Rap, 5 nM); valproic acid (VPA, 250 µm); tamoxifen (Tam, 1 µm); valproic acid and tamoxifen (VT); or valproic acid, rapamycin, and tamoxifen (VRT) for 48 h. **e** Representative western blot depicting S6RP, 4E-BP1, Histone H3, and ESR1 protein expression in MDA-MB-231 cells after combinational treatment with DMSO vehicle control; rapamycin (Rap, 5 nM); valproic acid (VPA, 250 µm); tamoxifen (Tam, 1 µm); valproic acid and tamoxifen (VT); or valproic acid, rapamycin, and tamoxifen (VRT) for 48 h. **f** Schematic depicting the proposed model for the combinational treatment (VRT). Rapamycin (Rap) effectively inhibits S6RP phosphorylation but upregulates 4E-BP1 phosphorylation, incapable of completely inhibiting mTORC1. Valproic acid inhibits HDAC expression and in combination with tamoxifen (VT) restores ESR1 expression and suppresses 4E-BP1 phosphorylation without affecting S6RP phosphorylation. Combination of VRT promotes ESR1 expression and H3 acetylation (i.e., suppressing HDAC) and suppresses both S6RP and 4E-BP1 (i.e., complete inhibition of mTORC1). **g**–**h** MTT viability analysis of SUM149-PT cells and MDA-MB-231 cells after 120 h of exposure to vehicle control DMSO (D); rapamycin (R, 5 nM); valproic acid (V, 250 µM); tamoxifen (T, 1 µM); valproic acid and tamoxifen (VT); or valproic acid, rapamycin, and tamoxifen (VRT). Data represents means ± SD, *n* = 3 for **a**–**h**; *p < 0.05, **p < 0.01

Rapamycin has been reported to partially inhibit mTORC1 signaling as it ineffectively inhibits 4E-BP1 phosphorylation^44^. Indeed, siRNA knockdown of S6RP or 5 nM rapamycin effectively suppressed S6RP phosphorylation but upregulated 4E-BP1 phosphorylation (Fig. 3a, b).

It has been shown that 4E-BP1 phosphorylation can be robustly stimulated by 17β-estradiol but inhibited by tamoxifen^45,46^. Suppressing HDACs has also been demonstrated to inhibit 4E-BP1 phosphorylation in a preclinical study^47^. As such, we sought to determine whether HDAC and tamoxifen together could inhibit 4E-BP1 as well as rapamycin-induced 4E-BP1 upregulation when in combination with rapamycin, leading to a complete mTORC1 inhibition. As expected, siRNA knockdown of S6RP in combination with 250 µM valproic acid and 1 µM tamoxifen effectively suppressed both S6RP and 4E-BP1 phosphorylation (Fig. 3c). Additionally, we found that the combination of 250 µM valproic acid and 1 µM tamoxifen, but neither alone, reproducibly restored ESR1 protein expression in TNBC cells (Fig. 3d).

For potential clinical application, we replaced S6RP siRNA with 5 nM rapamycin that showed a similar potency to siRNA knockdown in reducing S6RP phosphorylation. Consistently, 5 nM rapamycin, 250 µM valproic acid, and 1 µM tamoxifen (hereafter as VRT combination) restored ESR1 protein expression and inhibited both phosphorylated S6RP and 4E-BP1 proteins in TNBC cells (Fig. 3d, e). VRT combination also reduced cell viability of SUM149-PT and MDA-MB-231 TNBC cells (Fig. 3g, h). Notably, concentrations of tamoxifen, valproic acid, and rapamycin used in these experiments were clinically relevant, suggesting a tangible therapeutic approach to restore ESR1, inhibit mTORC1, and kill TNBC cells.

### VRT combination inhibits both non-CSC and CSC populations in the fractionated TNBC cells

The CSC subset (characterized by CD44^high/+^/CD24^low/−^) has been associated with chemoresistance and disease relapse. CSCs were capable of generating new tumors in mice with as few as 100 cells in comparison to non-CSC cells that required tens of thousands of cells^48^. In addition, chemotherapeutic drugs enriched CSCs after treatment. Thus the ability to inhibit both CSCs and non-CSCs and to reduce the conversion of non-CSCs to CSCs is instrumental for an effective treatment.

**Fig. 4:**
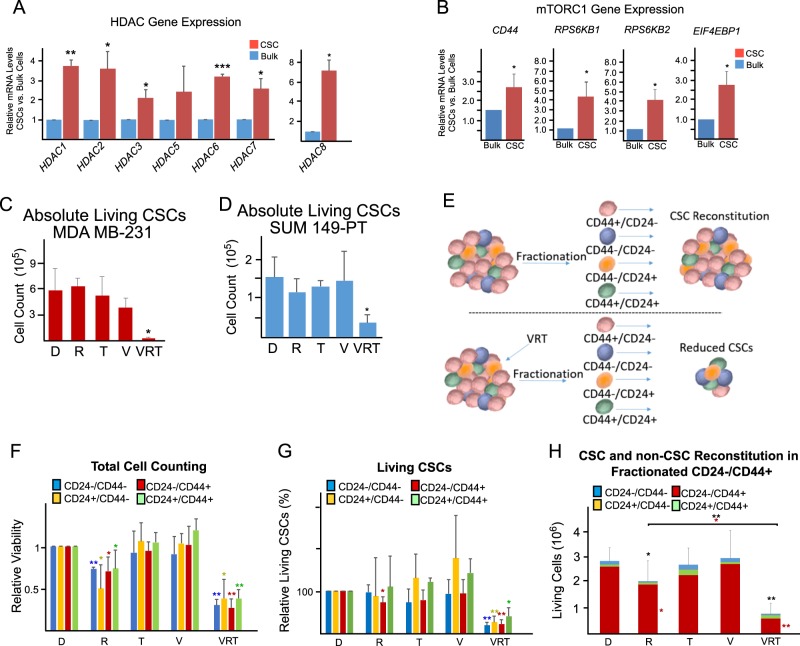
The gene expression levels of mTORC1 and HDACs are higher in TNBC CSCs than in non-CSCs; co-inhibition of mTORC1, ESR1, and HDACs suppresses the growth of both CSC and non-CSC subpopulations and promotes the conversion of CSCs to non-CSCs. **a**, **b** RT-qPCR analysis of the relative mRNA expression of HDAC and mTORC1 genes in fractionated MDA-MB-231 CSCs (CD44^high/+^/CD24^low/−^) and bulk populations after normalization with house-keeping gene *18s*. **c**, **d** Flow cytometric analysis of CD44^high/+^/CD24^low/−^ CSC subpopulation in SUM149-PT and MDA-MB-231 cells after 120 h of exposure to vehicle control DMSO (D); rapamycin (R, 5 nM); valproic acid (V, 250 µM); tamoxifen (T, 1 µM); or the combination of rapamycin, valproic acid, and tamoxifen (VRT). **e** MDA-MB-231 cells were fractionated into CSC (CD44^high/+^/CD24^low/−^) and non-CSC subpopulations based on CD44 and CD24 expression. Fractionated cells were exposed to vehicle (DMSO), rapamycin (5 nM), valproic acid (250 µM), and tamoxifen (1 µM) for 120 h. After treatment, fractionated cells were reanalyzed by flow cytometry to determine CSC and non-CSC subpopulations. **f** Fractionated MDA-MB-231 CSC and non-CSC subpopulations were treated as described in **e**. Cell viability was assessed by trypan-blue exclusion assays. **g** Relative living CSCs (CD44^high/+^/CD24^low/−^ and negative for both 7-AAD and Annexin-V staining) in each fractionated MDA-MB-231 subpopulation after treatments as described in **e**. **h** Fractionated MDA-MB-231 cells were treated as described in **e**. After assessment of cell viability with trypan-blue, the proportion of each subpopulation was determined by flow cytometry based on CD44 and CD24 expression. The total number of cells in each subpopulation was calculated: total viable cell number × the percentage of each subpopulation. Data represents means ± SD and *n* = 3 for **a**–**h**; *p < 0.05, **p < 0.01, ***p *<* 0.001

We fractionated MDA-MB-231 cells into CSC (based on CD44^high/+^/CD24^low/−^ expression) and three non-CSC subpopulations (CD44^high^/CD24^high^, CD44^low^/CD24^high^, and CD44^low^/CD24^low^) with >90% purity. RT-qPCR analysis revealed that HDAC- and mTORC1-related genes were expressed higher in CSCs than in non-CSCs (Fig. 4a, b). Significantly, VRT combination reduced the CSC subpopulation in MDA-MB-231 and SUM149-PT TNBC cells (Fig. 4c, d, Supplemental Fig. 2A). We further verified these results using siRNA knockdown of S6RP in combination with valproic acid and tamoxifen, showing a similar trend (Supplemental Fig. 3A-B).

VRT combination treatment reduced viability of all four fractionated subpopulations (i.e., CSCs and non-CSCs, Fig. 4e, f). We counted the total cell number and analyzed the percentage of each subpopulation within each fractionated subset based on CD44 and CD24 expression using flow cytometry after 120 h of treatment with VRT. Significantly, VRT combination treatment not only reduced living CSCs in each fractionated subpopulation but also diminished viability of non-CSCs in each subpopulation (Fig. 4g, Supplemental Fig. 2B). Furthermore, the remaining cells within each fractionated subpopulation after VRT combination treatment were shifted away from a CSC phenotype to non-CSC subpopulations (e.g., CD24^high^/CD44^low^, CD24^low^/CD44^low^, or CD24^high^/CD44^high^, Fig. 4h and Supplemental Fig. 4A-C). To estimate the conversion, we normalized the living cells after treatment and graphed the percentage of each subpopulation against total population (taken as 100%). There was an increase in non-CSC subsets than the CSC subset after VRT combination treatment in the fractionated subpopulations based on CD44/CD24 marker expression (Supplemental Fig. 5A-D). These data suggest that VRT combination treatment is an effective approach to target TNBC CSC subpopulation.

### VRT combination treatment retards tumor growth and inhibits CSC subpopulation and tumorigenesis in vivo

**Fig. 5:**
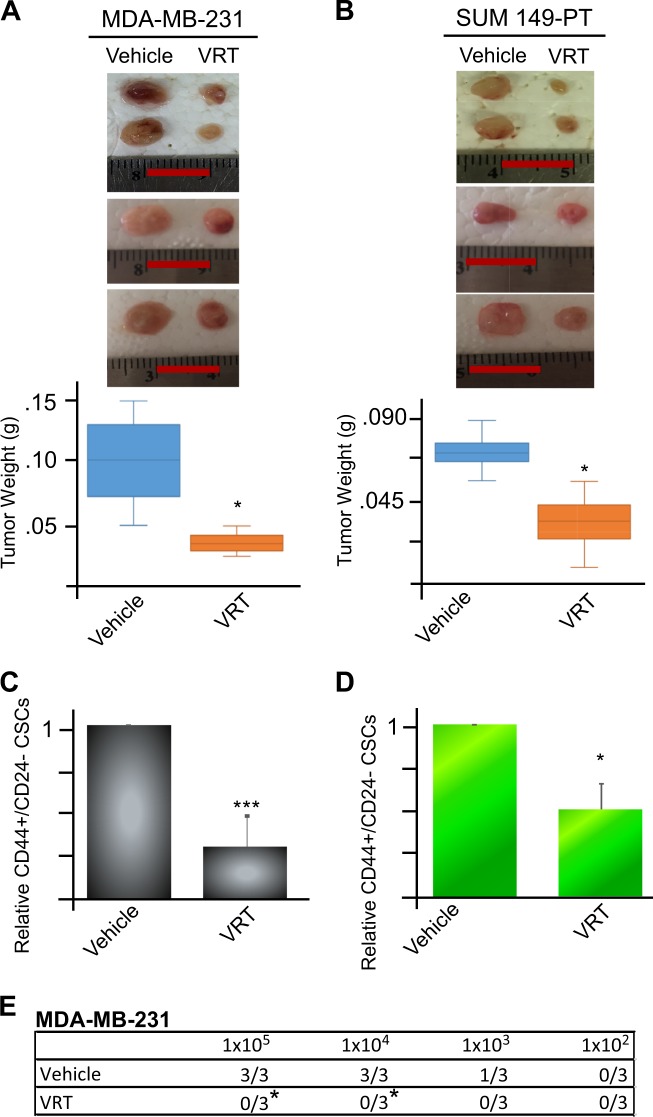
Co-inhibition of mTORC1, ESR1, and HDACs retards tumor growth and reduces CSCs and tumorigenesis in vivo. **a**, **b** MDA-MB-231 or SUM149-PT TNBC cells were injected into the mammary fat pads of athymic nude mice (2.5 × 10^6^ cells per fat pad). When the tumors reached a mean diameter of 3 mm, mice were randomly divided into two groups and intraperitoneally injected daily with vehicle (DMSO) or VRT combination (valproic acid, 300 mg/kg/day; rapamycin, 1.5 mg/kg/day, and tamoxifen, 0.4 mg/kg/day) for 20 days. The tumors were harvested, photographed, and weighed. Data represents means ± SD, *n* = 4, *p < 0.05. Scale bar = 0.5 cm. **c**, **d** Flow cytometric analysis of the CD44^high/+^/CD24^low/−^ CSC subpopulation in SUM149-PT and MDA-MB-231 cells dissociated from tumors after 20 days of treatment with the vehicle (DMSO) or VRT combination as described in **a**, **b**. Data represents means ± SD, *n* = 3, *p < 0.05, ***p < 0.001. **e** MDA-MB-231 tumors from **a**, **b** were dissociated into single-cell suspension and re-transplanted into the mammary fat pads of new athymic mice in serial dilutions (10^5^, 10^4^, 10^3^, 10^2^ cells per mammary pad per injection). Tumor formation was observed for 6 weeks. Data represents means ± SD, *n* = 3, *p < 0.05

We next determined the efficacy of VRT combination treatment in vivo. Since the combinations of valproic acid and rapamycin or valproic acid and tamoxifen showed less in vitro potent in inhibition of both CSCs and non-CSCs in comparison to VRT (data not shown), they were not included in the in vivo experiments. MDA-MB-231 and SUM149-PT TNBC cells were injected into the mammary fat pad of athymic mice. When tumor reached a mean diameter of 3 mm, mice were randomized into two groups and injected intraperitoneally with either vehicle (DMSO) or combination of valproic acid (300 mg/kg/day), rapamycin (1.5 mg/kg/day), and tamoxifen (0.4 mg/kg/day) for 20 days. As expected, VRT combination reduced tumor burden in both MDA-MB-231 and SUM 149-PT TNBC tumors (Fig. 5a, b).

At the end of the VRT combination treatment, we harvested and dissociated the tumors and assessed the CD44^high/+^/CD24^low/−^ subpopulation using flow cytometry. VRT combination treatment reduced CD44^high/+^/CD24^low/−^ CSC subpopulations in both MDA-MB-231 and SUM149-PT tumors in vivo (Fig. 5c, d, Supplemental Fig. 6).

To determine whether VRT combination inhibits tumor-initiating potential, we performed secondary transplantation. We serially diluted tumor cells containing various percentage of CD44^high/+^/CD24^low/−^ isolated from the primary tumors and transplanted them into athymic nude mice for 6 weeks without further treatment. MDA-MB-231 tumor cells from mice receiving VRT combination treatment exhibited diminished tumor-initiating capacity in comparison to the vehicle control (Fig. 5e). A similar trend was obtained from SUM149-PT tumor cells after secondary transplantation (Supplemental Figure 7). Thus VRT combination reduced tumor burden, suppressed CSCs, and tumorigenesis.

### TNBC patients’ tumors express similar levels of mTORC1 and HDAC to TNBC cell lines and VRT combination inhibits the growth of patients’ TNBC bulk and CSC populations

**Fig. 6:**
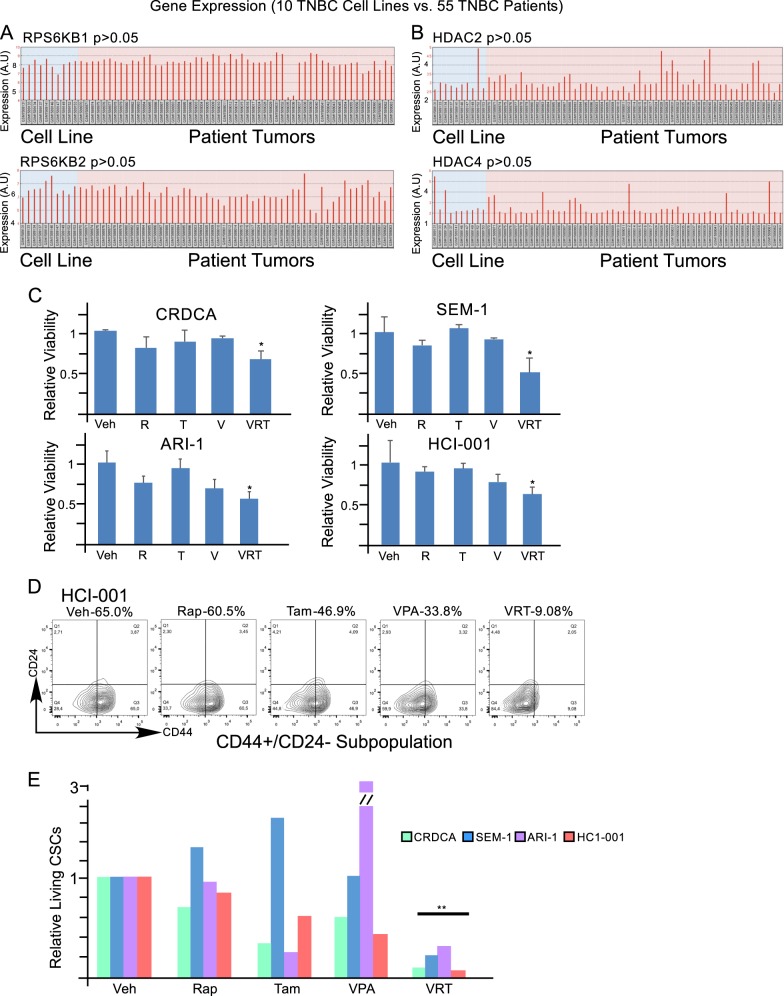
The expression levels of mTORC1 and HDAC are higher in TNBC cell lines and TNBC patient tumors; co-inhibition of mTORC1, ESR1α, and HDACs reduces the viability of patient tumors’ fragments and CSCs. **a**, **b** The expression of mTORC1 and HDAC genes in 10 TNBC cell lines and 55 TNBC patient samples were compared using the NCBI Gene Expression Omnibus (GEO2R). The GSE65216 samples were analyzed with the Affymetrix Human Genome U133 Plus 2.0 Array (GPL570). **c** Alamar blue viability analysis of three primary TNBC patient fragments (CRDCA, SEM-1, and ARI-1) and one patient-derived xenograft fragment (HCI-001). TNBC fragments were cultured and treated for 144 h with vehicle (DMSO, D), rapamycin (5 nM, R), tamoxifen (1 µM, T), valproic acid (250 µM, V), or VRT combination. **d** Representative flow cytometric data showing the percentages of CSC (CD44^high/+^/CD24^low/−^) subpopulation in patient-derived xenograft TNBC fragments after treatments as described in **c**. **e** Relative living CSCs (CD44^+/high^/CD24^−^^/low^ and also negative for 7-AAD and Annexin-V) in TNBC patient tumor fragments after treatment as described in **c**

In comparison to TNBC cell lines (10 samples), 55 primary TNBC patient samples expressed similar levels of mTORC1 and HDAC2 and HDAC4 (omnibus2R platform Dataset: GSE65216, Accessed November 1 2017^39–43^, Fig. 6a, b). VRT combination treatment suppressed viability of primary TNBC patients’ tumor slices (CRDCA, SEM-1, and ARI-1) and a patient-derived xenograft tumor slices (HCI-001) (Fig. 6c)^49^. Furthermore, VRT combination treatment reduced CD44^high/+^/CD24^low/−^ CSC subpopulation (Fig. 6d, e). Together, these results indicate that co-inhibition of mTORC1, HDAC, and ESR1 can be considered as a potential treatment for patients with TNBC.

## Discussion

Anti-estrogen therapies have been used for the treatment of ESR1-positive breast cancers due to its excellent efficacy-to-toxicity ratio. Since TNBC does not possess targetable markers, functional activation of ESR1 expression, via inhibition of HDACs and mTORC1 to render TNBC sensitive to endocrine treatment, has been an attractive approach^50–52^.

HDACs interact with and repress ESR1 at multiple levels along the ESR1 pathway^27,28^. A HDAC inhibitor Z-ligustilide was shown to restore ESR1 protein expression in ESR1-negative breast cancer lines, re-sensitizing cells to tamoxifen^53^. Treatment with HDAC inhibitor Trichostatin A was shown to restore ESR1 gene and protein expression in ESR1-negative breast cancer^54^. The HDAC inhibitor vorinostat was also tested to upregulate ESR1 in TNBC cells^55^.

However, contrasting results showed that HDAC inhibition does not induce ESR1 gene expression in TNBC and even repress ESR1 in luminal breast cancer under certain conditions^33,56^. We also found that HDACs’ inhibitor valproic acid alone was not able to restore ESR1 protein expression in TNBC cells. However, valproic acid in combination with the mTORC1 inhibitor rapamycin reproducibly enhanced ESR1 protein expression in TNBC cells. By analysis of clinical datasets, we found that TNBC expressed high levels of HDAC and mTORC1 in comparison to non-TNBC luminal breast cancers. Additionally, the level of mTORC1 expression is positively correlated with that of HDAC expression in TNBC patients’ samples. Thus repressed ESR1 in TNBC could be partially attributable to dual activation of mTORC1 and HDACs.

HDAC5 has been shown to co-precipitates with regulatory-associated protein of mTOR (Raptor); HDAC5 inhibition promotes Raptor acetylation, subsequently inhibiting mTORC1 signaling^57^. Conversely, P13K/Akt/mTOR regulates HDAC3 phosphorylation, promoting its activity^58^. This suggests that mTORC1 facilitates HDAC expression and vice versa, providing a rationale for using valproic acid and rapamycin to promote histone H3 acetylation and ESR1 re-expression, as shown in this report.

Previous studies showed that inhibition of P13K/Akt/mTORC1 signaling alone was ineffective in sensitizing ESR1-positive or -negative breast cancer to endocrine therapy^14^. The ineffectiveness of mTORC1 inhibitors in tumor treatment^23–25^ and in functional reactivation of ESR1 may be related to incomplete inhibition of 4E-BP1 phosphorylation, because rapamycin was known to potently inhibit phosphorylation of S6RP but not that of 4E-BP1^26,44,59,60^. Thus inhibition of 4E-BP1 phosphorylation by rapamycin was transient (within 6 h) and afterwards became resistant to rapamycin treatment^26^. As a result, cap-dependant translation via mTORC1 signaling can be maintained in the presence of rapamycin. Consistently, retrospective studies of 93 breast cancer patients showed that elevated 4E-BP1 protein was associated with a poor response to endocrine treatment^61,62^.

In this report, we found that the combination of valproic acid and tamoxifen is capable of inhibition of 4E-BP1 phosphorylation, which is associated with functional restoration of ESR1 TNBC. It has been reported that HDAC2 promotes eIF4E/4E-BP1 signaling and cap-dependant translation^63^, which can be inhibited by valproic acid. Similarly, tamoxifen has been found to inhibit 4E-BP1 in a MDA-MB-231 tumor xenograft through an ER-independent mechanism^64^. Tamoxifen has also been shown to modify histone activity^61,65^. It is of note that treatment with tamoxifen alone or in combination with rapamycin resulted in enhanced 4E-BP1 phosphorylation in ESR1-positive breast cancer cell lines^62^. Mechanisms by which tamoxifen plus valproic acid (HDAC inhibitor), but not tamoxifen plus rapamycin (mTORC1 inhibitor), could effectively prevent 4E-BP1 phosphorylation and restore functional ESR1 expression remain to be further defined.

Cancer cell plasticity^10,13^ is a big challenge. For an effective treatment, both CSC and non-CSC subpopulations should be concurrently targeted as bulk cancer cells (i.e., non-CSCs) are capable of converting into CSCs under certain conditions^10,66^. It has been reported that CSCs from patient tumor samples express high levels of S6RP and 4E-BP1 proteins^62,67^. We also observed that the fractionated CD44^high/+^/CD24^low/−^ CSCs expressed higher levels of S6RP and 4E-BP1 genes than their non-CSC counterparts. It seems that inhibition of both S6RP and 4E-BP1 in breast cancer is required for the suppression of CSCs. VRT combination treatment simultaneously inhibits both S6RP and 4E-BP1 and functionally activates ESR1 expression to re-sensitize TNBC cells for endocrine therapy. This might be one of the underlying mechanisms by which VRT combination treatment suppresses the growth of both TNBC CSCs and non-CSCs, thus reducing CSC enrichment from the fractionated non-CSC subpopulations.

In vivo, VRT combination treatment is also able to reduce tumor burden, inhibit CSCs, and diminish tumorigenicity after secondary transplantation. As valproic acid, tamoxifen, and rapamycin have been commonly used in the clinic, this study may lead to a new, clinically translatable approach for TNBC treatment.

## Electronic supplementary material

Supplemental Figures 1-7

Supplementary figure legends
