# Supplementary material for: Co-inhibition of mTORC1, HDAC and ESR1α retards the growth of triple-negative breast cancer and suppresses cancer stem cells
Source: Cell Death Dis. 2018 Jul 26;9(8):815. doi: 10.1038/s41419-018-0811-7 (PMC6062597; doi:10.1038/s41419-018-0811-7)
Supplement: Supplementary file 1 — Supplemental Figures 1-7 [file 41419_2018_811_MOESM1_ESM.pdf]

Supplemental Figure 1

A

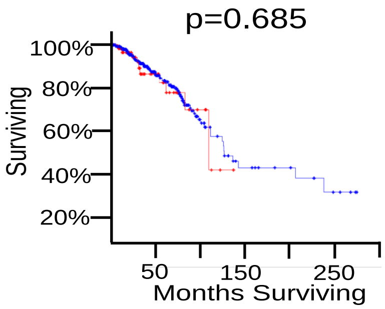

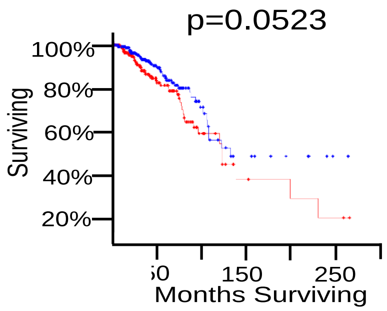

	HDAC Expression	
	High	Low
Total Cases	72	454
Deceased	11	72
Median Months Survival	112.2	129.6

	mTORC1 Expression	
	High	Low
Total Cases	246	281
Deceased	45	38
Median Months Survival	129.4	140.1

Supplemental Figure 2

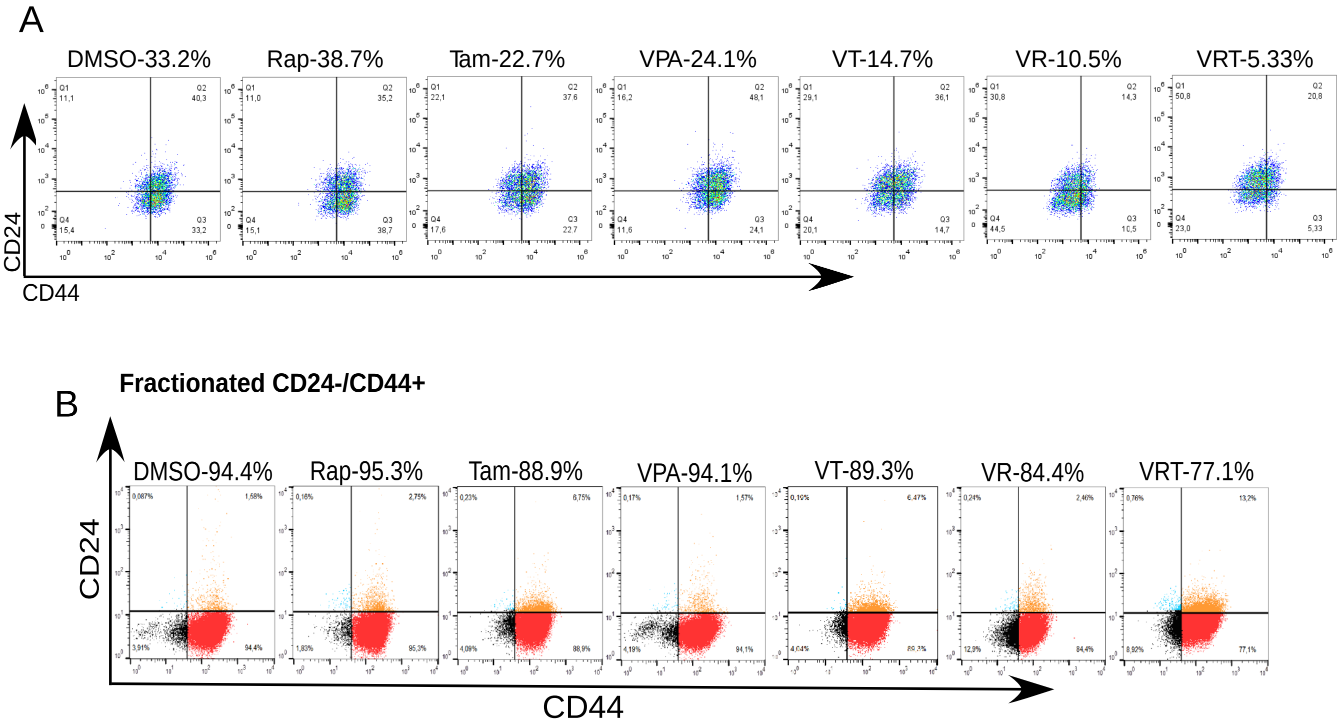

Supplemental Figure 3

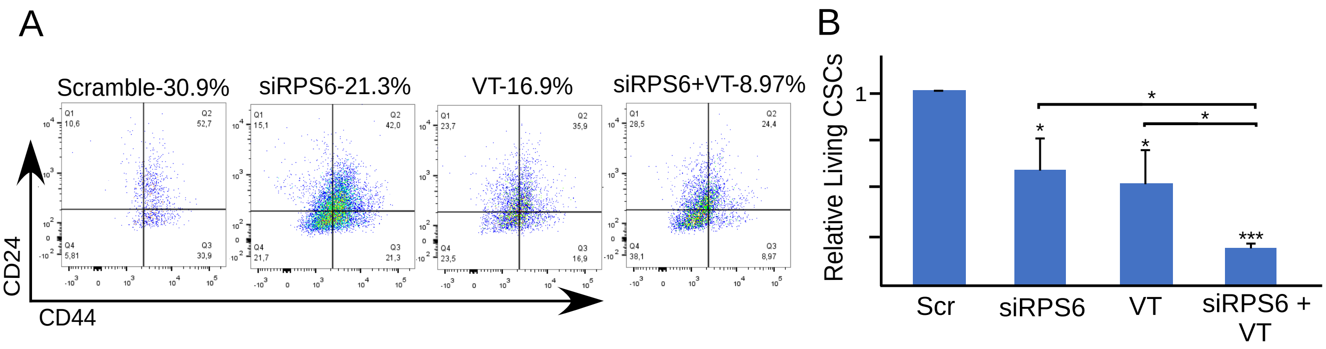

Supplemental Figure 4

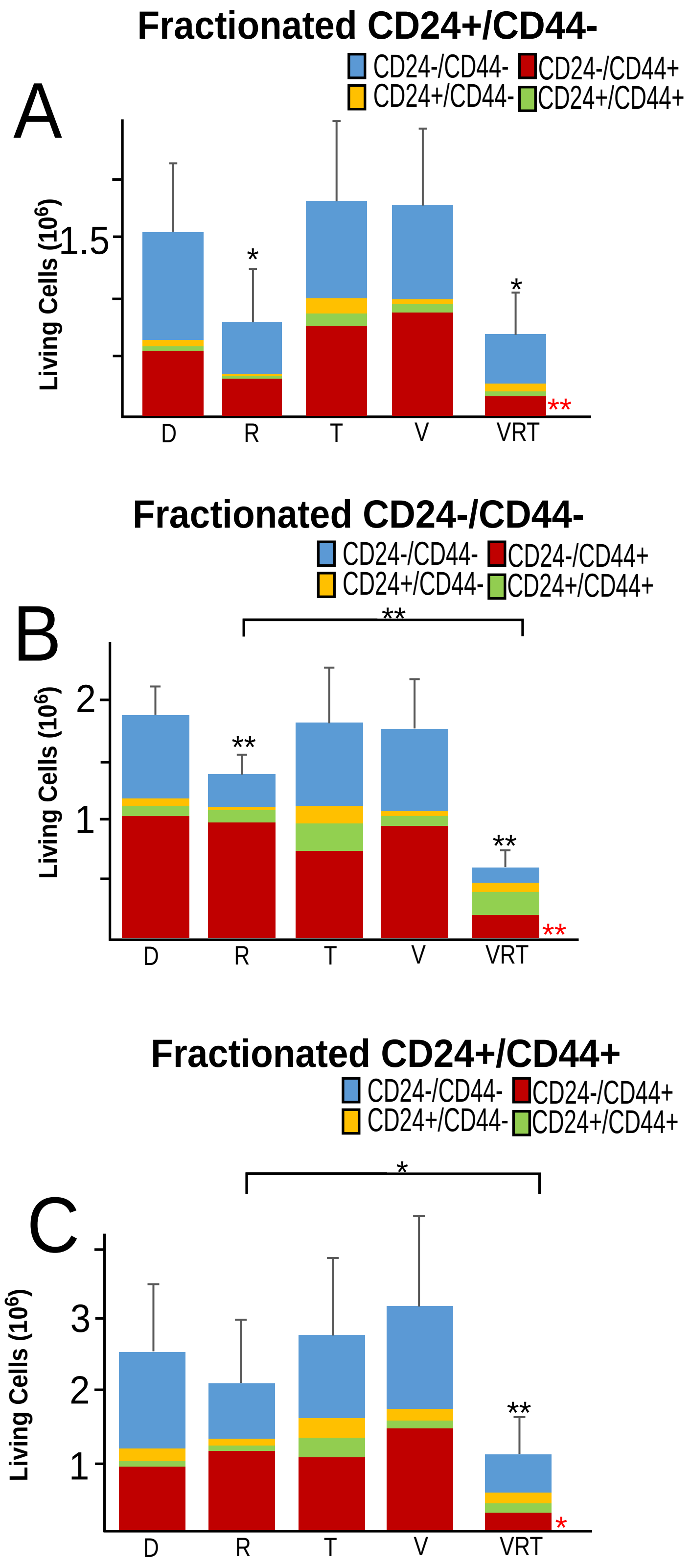

Supplemental Figure 5

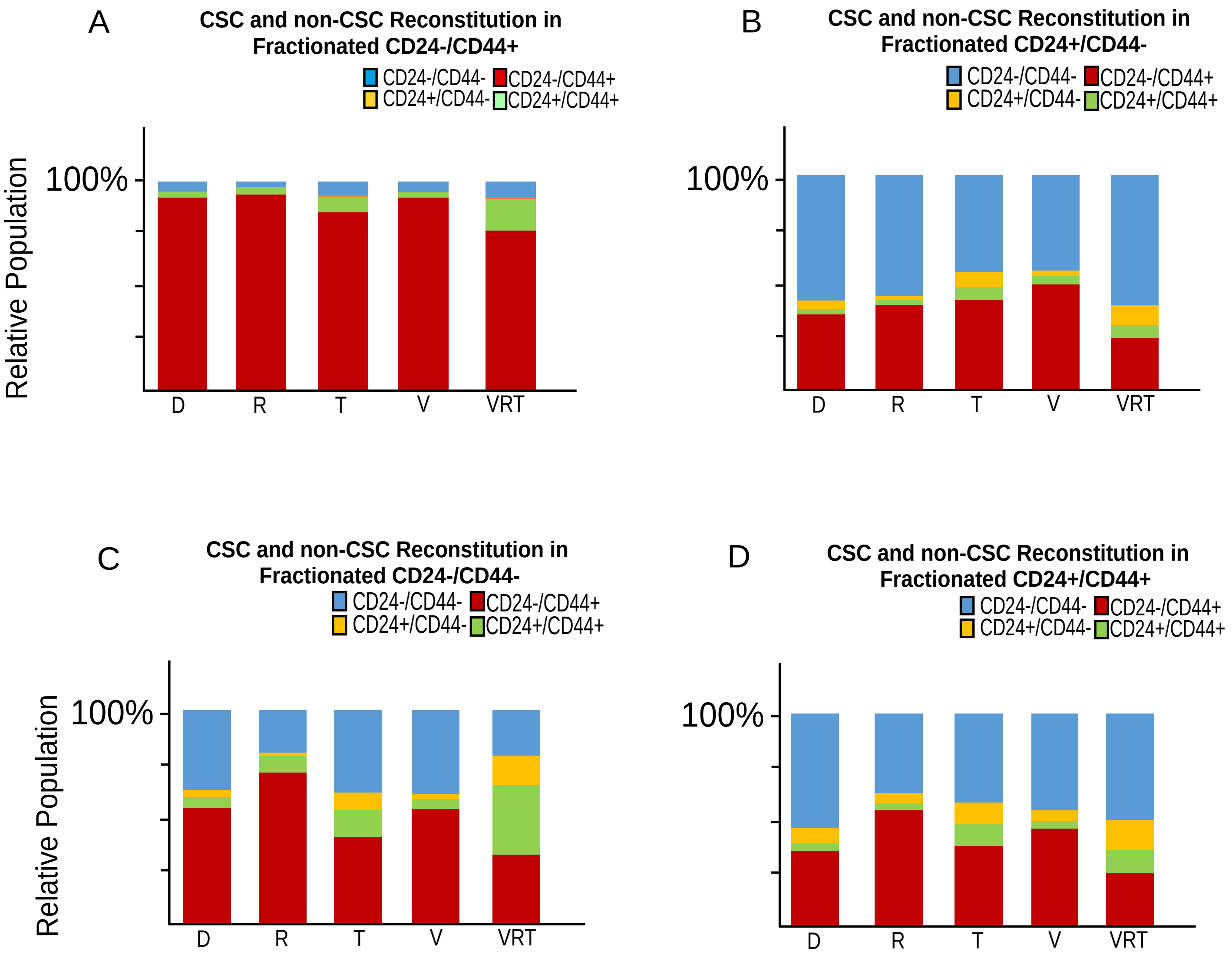

Supplemental Figure 6

A

MDA MB-231 Xenograft

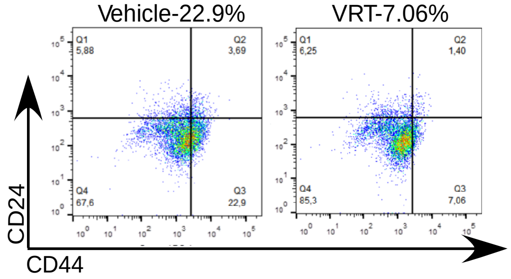

B

SUM 149-PT Xenograft

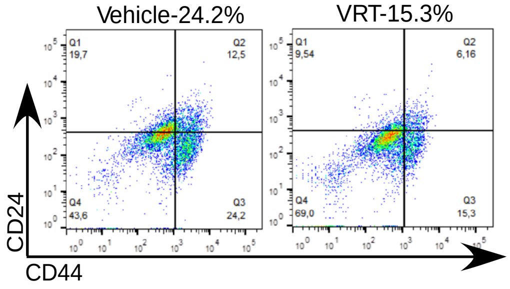

Supplemental Figure 7

A

SUM 149-PT

	$1x10^5$	$1x10^4$	$1x10^3$	$1x10^2$
Vehicle	3/3	2/3	1/3	0/3
VRT	2/3	0/3	0/3	0/3

Table S1. Primers used in RT-qPCR

Genes	Forward	Reverse
**18S**	AACCCGTTGAACCCCATT	CCATCCAATCGGTAGTAGCG
**HDAC1**	ACCGGGCAACGTTACGAAT	CTATCAAAGGACACGCCAAGTG
**HDAC2**	TCATTGGAAAATTGACAGCATAGT	CATGGTGATGGTGTTGAAGAAG
**HDAC3**	TTGAGTTCTGCTCGCGTTACA	CCCAGTTAATGGCAATATCACAGAT
**HDAC5**	TTGGAGACGTGGAGTACCTTACAG	GACTAGGACCACATCAGGTGAGAAC
**HDAC6**	TGGCTATTGCATGTTCAACCA	GTCGAAGGTGAACTGTGTTCCT
**HDAC7**	CTGCATTGGAGGAATGAAGCT	CTGGCACAGCGGATGTTTG
**HDAC8**	TCCCGAGTATGTCAGTATATATGA	GCTTCAATCAAAGAATGCACCAT
**RPS6KB1**	CACATAACCTGTGGTCTGTTGCTG	AGATGCAAAGCGAACTTGGGATA
**RPS6KB2**	CTTCCAGACTGGTGGCAAACTCTA	CAGCGTGATCTCAGCCAGGTA
**EIF4EBP1**	CCCGCTTATCTTCTGGGCTA	CTATGACCGGAAATTCCTGATGG
**CD44**	AGACAACCACAAGGATGACTGATG	TCCAGTTTCCTTCATAAGCAGTGG
**GAPDH**	ACAGTCAGCCGCATCTTCTT	GACAAGCTTCCCGTTCTCAG
**ESR1**	GGAGACATGAGAGCTGCCAAC	CCAGCAGCATGTCGAAGATC
