# Supplementary material for: Co-inhibition of mTORC1, HDAC and ESR1α retards the growth of triple-negative breast cancer and suppresses cancer stem cells
Source: Cell Death Dis. 2018 Jul 26;9(8):815. doi: 10.1038/s41419-018-0811-7 (PMC6062597; doi:10.1038/s41419-018-0811-7)
Supplement: Supplementary file 2 — Supplementary figure legends [file 41419_2018_811_MOESM2_ESM.docx]

**Supplemental Figure 1: Kaplan-Meier survival curve for patients with invasive breast cancer with upregulated mTORC1 or HDAC in tumor samples.**

**(A-B)** Kaplan-Meier survival curve for survival of the patients with high level expression of mTORC1 or HDAC genes in cancer samples (red curve) in comparison to the patients with unaltered expression (blue curve). N = 527, * *p* < 0.05, log-rank test.

**Supplemental Figure 2: Combinational inhibition of mTORC1, ESR1 and HDAC using rapamycin, valproic acid and tamoxifen suppresses CSCs in TNBC cells.**

**(A)** Representative flow cytometric data showing percentages of CSC (CD44^high/+^/CD24^low/-^) subpopulation in MDA-MB-231 cells after 120 hours of treatment with vehicle (DMSO), valproic acid (250 µM, VPA), rapamycin (5 nM, Rap), tamoxifen (1 µM, T) or VT (VPA+T), or VRT (VPA+Rap+T) combination.

**(B)** Representative flow cytometric analysis of CD44^high/+^/CD24^low/-^ CSC subpopulation in the fractionated CD44^high/+^/CD24^low/-^ MDA-MB-231 cells after 120 hours of treatments as described in A.

**Supplemental Figure 3: S6RP knockdown in combination with valproic acid and tamoxifen inhibits CSCs in TNBC cells.**

**(A-B)** Representative and tabulated flow cytometric data showing percentages of CSC (CD44^high/+^/CD24^low/-^) subpopulation in MDA-MB-231 cells after 72 hours of treatment with scramble control, siRNA knockdown of S6RP in combination with tamoxifen (1 µM, T) and valproic acid (250 µM, VPA).

**Supplemental Figure 4: Rapamycin in combination with valproic acid and tamoxifen reduces CSC enrichment in the fractionated non-CSC subpopulations.**

**(A-C)** Fractionated MDA-MB-231 non-CSC subpopulations were exposed for 120 hours to vehicle, valproic acid (250 µM, VPA), rapamycin (5 nM, Rap), and tamoxifen (1 µM, T). After treatment, cell viability was determined by trypan-blue exclusion assay and the proportion of each non-CSC and CSC subpopulations was determined by flow cytometry based on CD44 and CD24 expression. The total number of cells in each subpopulation was calculated using total viable cell numbers × percentage of each subpopulation.

**Supplemental Figure 5: Rapamycin in combination with valproic acid and tamoxifen reduces CSC conversion from non-CSC in the fractionated CSCs and non-CSC subpopulations.**

**(A-D)** Fractionated MDA-MB-231 CSC and non-CSC subpopulations were exposed for 120 hours to VRT combination as described in Figure 4. After treatment, cell viability was assessed by trypan-blue exclusion assay and the proportion of each CSC and non-CSC subpopulation was determined by flow cytometry based on CD44 and CD24 expression. The total number of cells in each subpopulation was calculated using viable cell number × percentage of each subpopulation. The populations were then normalized to estimate the reconstitution of CSC from each non-CSC subpopulation after VRT combination treatment.

**Supplemental Figure 6: Treatment with rapamycin in combination with valproic acid and tamoxifen *in vivo* reduces CSCs in TNBC tumors *in vivo*.**

**(A-B)** Representative flow cytometric data showing percentages of CSC (CD44^high/+^/CD24^low/-^) subpopulation in dissociated cells from MDA-MB-231 and SUM 149-PT tumors after 20 days of treatment with the vehicle (DMSO) or VRT combination as described in Figure 5.

**Supplemental Figure 7: Co-inhibition of mTORC1, ESR1 and HDACs showed a trend to reduce tumorigenesis of TNBC SUM149-PT cells.**

SUM149-PT tumors from Figure 5B were dissociated into single cell suspension and re-transplanted into the mammary fat pads of new athymic mice in serial dilutions (10^5^, 10^4^, 10^3^, 10^2^ cells per mammary pad per injection) without further treatment. Tumor formation was observed for 6 weeks. Treatment with rapamycin in combination with valproic acid and tamoxifen reduced tuomorigenesis although a statistically significance was not achieved.
